# Current State of Immunotherapy for Treatment of Glioblastoma

**DOI:** 10.1007/s11864-019-0619-4

**Published:** 2019-02-21

**Authors:** Tresa McGranahan, Kate Elizabeth Therkelsen, Sarah Ahmad, Seema Nagpal

**Affiliations:** 1 Department of Neurology, UW Medicine, University of Washington, Seattle, WA USA; 2 Department of Neurology, Stanford University, Stanford, CA USA

**Keywords:** Glioblastoma, Immunotherapy, Vaccine, CAR-T, Checkpoint inhibitors

## Abstract

At this time, there are no FDA-approved immune therapies for glioblastoma (GBM) despite many unique therapies currently in clinical trials. GBM is a highly immunosuppressive tumor and there are limitations to a safe immune response in the central nervous system. To date, there have been several failures of phase 3 immune therapy clinical trials in GBM. These trials have targeted single components of an antitumor immune response. Learning from these failures, the future of immunotherapy for GBM appears most hopeful for combination of immune therapies to overcome the profound immunosuppression of this disease. Understanding biomarkers for appropriate patient selection as well as tumor progression are necessary for implementation of immunotherapy for GBM

## Introduction

Glioblastoma (GBM) is the most common and most aggressive primary malignant brain tumor in adults. Clinical trials in GBM have led to incremental improved median overall survival (mOS) that have improved survival in the general GBM population [1•]. While the most recent positive phase 3 clinical trial of tumor treating fields (TTF) had a mOS of 20.5 months [2••], it is the tail of long-term survivors that continues to provide hope to patients and neuro-oncologists. TTF gained acceptance not just because of a significant improvement in survival, but an increase in 5-year survival to 13% compared to 5% in the standard of care (SOC) arm [2••]. Durable responses to immunotherapy, seen in many cancers, have re-invigorated the study of immunotherapy in GBM.

The brain is not as immuno-privileged as once thought, yet obstacles remain for immunotherapy in treating GBM. Malignant gliomas are one of the most immunosuppressive solid tumors due in part to lymphopenia driven by bone marrow suppression [3]. GBM is also an immunologically quiet tumor, with low tumor mutational burden (TMB), few tumor infiltrating T cells (TILS), and low PD-1/PD-L1 expression, especially when compared to other cancers where immunotherapy has been the most successful [4, 5]. GBM is also a profoundly heterogeneous tumor which facilitates immune evasion [6]. The SOC for newly diagnosed GBM, the combination of radiation therapy (RT) and alkylating chemotherapy, confounds this immunosuppression. Often steroids are necessary for management of peritumoral edema but they decrease the efficacy of immunotherapies [7, 8]. In addition to hurdles for stimulating an effective immune response against GBM, robust immune activation within the intracranial space poses clinical safety risks including complications of cytokine release syndrome and autoimmune encephalitis [9–11]. Despite these challenges, long-term responders to immunotherapy have been reported in clinical trials of GBM, but no predictive biomarkers exist at this time [12•].

Immunotherapy in GBM has a long history, including immune stimulation, antibody-mediated immunotherapies, adoptive cellular immunotherapies, and vaccines [13]. At this time, phase 3 clinical trials have not demonstrated efficacy for immunotherapy in GBM and no FDA-approved immunotherapy for GBM exists. Given the challenges of immunotherapy for GBM, a combination approach will likely be required. Additionally, identification of biomarkers for patient selection and disease surveillance are essential. Given the risks of recurrent tumor sampling, there is a search for serum, CSF, and imaging biomarkers for GBM.

Here, we discuss recent findings and ongoing clinical research into immunotherapy for GBM, including checkpoint inhibitors, vaccines, CAR-T therapy, and viral therapy. We will also discuss our current understanding of biomarkers in GBM that influence candidacy for clinical trials and may explain the response and failures to different immune therapies.

## Checkpoints

Checkpoint inhibition has revolutionized treatment of several advanced malignancies providing hope for cancer treatment and resulting in a well-deserved Nobel Prize. Inhibitors of immune checkpoints such as PD-1, PD-L1, and CTLA-4 promote a shift from the normal balance of the adaptive immune system to increased immune activation [14]. In preclinical data, checkpoint inhibitors showed promise for treatment of GBM; however, translation of this preclinical work to patients was complicated by CNS toxicity when used to treat other malignancies [11, 15].

An early phase 1 study of nivolumab (nivo) alone or in combination with ipilimumab (ipi) for treatment of recurrent GBM (rGBM) found higher toxicity with the combination PD-1 and CTLA-4 inhibition, but comparable mOS (12-month mOS 40% v. 30%) [16]. Based on the tolerance of nivo monotherapy and promising mOS compared to historical controls, a large phase 3 trial was developed. CheckMate-143 randomized patients with rGBM to treatment with nivo or bevacizumab (BEV). The genotoxic stress of radiation and chemotherapy used in SOC treatment for newly diagnosed GBM were predicted to increase the TMB in rGBM and favor response to checkpoint inhibition. Unfortunately, at interim analysis of 369 patients, nivo did not demonstrate a mOS benefit over BEV (9.8 m nivo v. 10 m BEV) [17]. It should be noted however that for patients who did respond, those responses were more durable in the nivo arm (11.1 m nivo v 5.3 BEV), again raising enthusiasm for a tail of long term survivors [16]. A single center retrospective of the use of pembrolizumab (pembro) in rGBM also found no benefit [18]. Randomized controlled clinical trials examining the use of nivo in the setting of newly diagnosed GBM (CheckMate 498 and 548) are ongoing. Checkpoint inhibition for GBM was recently reviewed in this journal [19].

Promising data from NSCLC, head and neck cancer, and bladder cancer has stimulated interest in neoadjuvant checkpoint inhibition in GBM [20, 21]. Reported at Society for NeuroOnclogy 2018, patients receiving two doses of pembro prior to re-resection of rGBM had improved mOS (13.7 m neoadjuvant v. 7.5 m adjuvant only) compared to those starting pembro after re-resection [22]. While a small study, this work raises hope that the combination of pembro with surgery may help increase antigen exposure as well as TIL infiltration into rGBM.

Several other phase 1 and 2 clinical trials are currently examining the role of checkpoint inhibitors in combination with other therapies (Table 1). There is great interest in the combination of radiation with checkpoint inhibition, based partly on the idea that radiation may increase antigen presentation as well as promote the abscopal effect. These clinical trials are examining use in both newly diagnosed GBM with standard fractionated chemoradiation, as well as in the rGBM with radiosurgery and hypo-fractioned radiation. There is also a phase 1 study examining use of pembro with MRI-guided laser ablation for rGBM. Addition of pembro to the SOC including TTF is also being studied in the 2-THE-TOP phase 2 clinical trial (NCT03405792). Checkpoint inhibition with combination vaccines and oncolytic viruses will be discussed below.

**Table 1:** Combination Checkpoint Inhibition Trials in GBM

		Checkpoint inhibitor	Additional therapy	Phase	Clinical trials
Dual Checkpoint blockade	New diagnosis GBM	CTLA-4 (ipilimumab)	PD-1 (nivolumab)	I	NCT02311920
	rGBM	PD-1 (nivolumab)	Anti-LAG-3(BMS 986016) or anti CD137(urelumab)	I	NCT02658981
	refractory solid tumors	PD-1 (nivolumab)	anti-CD-27 (varlilumab)	I/II	NCT02335918
	Advanced refractory cancers	PD-1 (nivolumab)	Intratumoral IDO1 inhibitor (INT230-6)	I/II	NCT03058289
	Advanced cancers	PD-1 (nivolumab)	IDO1 inhibitor (epacadostat)	I/II	NCT02327078
	rHGG	PD-L1 (durvalumab)	CTLA-4 (tremelimumab)	II	NCT02794883
Vaccines	New diagnosis GBM	PD-1 (pembrolizumab)	HSPPC-96	II	NCT03018288 AVeRT
	rHGG	PD-1 (nivolumab)	pp65 DC	I	NCT02529072
	rGBM	PD-1 (nivolumab)	DCVAX-L	II	NCT03014804
Oncolytic virus	rGBM	PD-1 (pembrolizumab)	DNX-2401	II	NCT02798406
Radiation	rHGG	pembro	hypofractionated stereotactic irradiation	I	NCT02313272
	rGBM	nivo	SRS + Valproic acid	I	NCT02648633
	rHGG	nivo	hypofractionated stereotactic irradiation	I	NCT02829931
	rGBM	PD-L1 (durvalumab)	hypofractionated stereotactic irradiation	I/II	STERIMGLI - NCT02866747
Laser ablation	rHGG	MK-3475	MRI-guided laser ablation	I/II	NCT02311582
CSF-1R inibition	rHGG	nivo	CSF-1r inhibitor (BLZ945)	I/II	NCT02526017
	rGBM	PD-1 (PDR001)	CSF-1r inhibitor (FPA008)	I	NCT02829723
TGF-beta	advanced solid tumors	nivo	TGF-beta inhibiotr (Galunisertib)	I/II	NCT02423343

*rHGG* recurrent high grade glioma, *rGBM* recurrent glioblastoma, *SRS* stereotactic radiosurgery

Preclinical research suggested that low-dose VEGF inhibition promotes the switch in the tumor microenvironment from immunosuppressive (M2-like) to a more immune supportive (M1-like) tumor microenvironment [23]. In melanoma, the combination of CTLA-4 inhibition (ipi) with VEGF inhibition of BEV found disease control rate of 67.4% in 46 patients treated [24]. Aside from the change in the tumor microenvironment, BEV may play another important role in GBM patients as a steroid substitute [25, 26]. A recent retrospective study of non-small cell lung cancer (NSCLC) patients demonstrated a greater than 10% reduction in overall response rate (complete and partial responses) in patients who were on any dose of steroid greater than 10 mg of prednisone a day (= 1.6 mg of dexamethasone) prior to starting treatment with a checkpoint inhibitor. This baseline steroid use was significantly associated with decreased PFS and mOS [8]. This raises concern that necessary management of peritumoral edema in GBM patients, even with the minimal effective dose of dexamethasone needed to control symptoms, may be sufficient to dampen response to checkpoint inhibition and possibly other immunotherapy. In GBM, BEV can be used as a steroid substitute and the safety of the combination of BEV with checkpoint inhibition may provide an opportunity to treat peritumoral edema without the immunosuppressive effects of steroids. For rGBM, there are several ongoing clinical trials examining PD-1 and PD-L1 inhibitors in combination with BEV. Preliminary results from these studies support the safety of this combination; however, among rGBM patients, the combination of pembro with BEV does not improve survival [27].

At this point, there is no role for checkpoint inhibition monotherapy in the treatment of most patients with GBM; however, the combination of checkpoint inhibition with other immune stimulating therapies may be considered. Serious, and even fatal, CNS immune adverse events have been reported with checkpoint inhibition [11]. Given this risk with checkpoint inhibitor monotherapy, as therapies seek to increase immune activation against GBM, there remains concern for complications for over activation of the immune system within the brain.

### Checkpoint biomarkers

In parallel with these therapeutic trials, there are several ongoing studies to help better understand biomarkers to predict response to checkpoint inhibition. A number of biomarkers are thought to predict response to PD-1 and PD-L1 inhibitors in other malignancies. Specifically in NSCLC, it has become increasingly clear that response to PD-1 inhibitors correlates with the level of PD-L1 expression in tumor. In Keynote-042, a study of pembro compared to platinum-based chemotherapy in first-line metastatic NSCLC, patients with high expression (> 50%) receiving pembro had a 20.0-month mOS compared to 12.2 months in the chemotherapy group (HR 0.69). In contrast, patients with expression between 1 and 49% receiving pembro had a mOS 13.4 months versus 12.1 months with chemotherapy (HR 0.92) [28]. The CheckMate-057 study of nivo monotherapy versus docetaxel demonstrated no benefit for checkpoint inhibition in tumors with < 1% PD-L1 expression [29]. A study of 94 patients with GBM found median PD-L1 expressional 2.77% and that PD-L1 expression correlated with worse outcome [30] while an earlier study did not find PD-L1 to be a negative prognostic factor [31]. The role of PD-L1 expression on GBM tumor cell in response to checkpoint inhibition is unclear.

To better understand changes in the tumor microenvironment with PD-1 inhibition, pembro was given prior to re-resection in patients with GBM (NCT02337686). Analysis of the resected tumor demonstrated low T cell infiltrate that was not modulated by PD-1 inhibition [32]. Of note, while use of pembro did not improve survival, all patients required steroids after pembro. Studies of neoadjuvant checkpoint inhibition have found a trend toward increased TIL fractions as well as changes in several immune markers [22].

Two tests which reflect the overall genetic stability of tumors, TMB and microsatellite instability (MSI), also play a role in predicting which patients will have meaningful responses to PD-1 axis drugs. In 2017, pembro was approved for patients with MSI or mismatch repair deficiencies for all solid tumors regardless of histology. Higher TMB and MSI correlate with longer mOS [33]. In glioma patients, favorable status across all three of these biomarkers (PD-L1, MSI, TMB) appears to be rare and suggest that only a minority of patients will respond to checkpoint monotherapy [34]. Low frequency of these markers in GBM may be contributing to the disappointing results of PD-1/PD-L1 monotherapies to date [35].

## Vaccines

Cancer vaccine therapy in GBM is not preventative, but rather is designed to induce an immune response against the tumor. For GBM, vaccines encompass a range of therapies including direct exposure to antigens (peptide or DNA) in combination with immune-stimulating molecules as well as stimulated patient-derived antigen presenting cells (dendritic cells (DC)). GBM antigen targets are most often tumor-associated antigens given GBM-specific antigens are rare. Some of these antigens are restricted by HLA types, limiting the patient population in which these vaccines may be considered. Use of whole tumor lysate as an antigen was lethal when studied in animal models [36]; however, modifications to GBM tumor lysate, such as heat shock proteins (HSP) and DC vaccines, have been well tolerated with promising early results. Tumor antigen vaccines and customized vaccines will be discussed separately below.

### Tumor antigen vaccines

Cancer tumor antigens may be tumor-specific or tumor-associated antigens. At this time, the best studied tumor-specific antigen is a constitutively activated mutation of epidermal growth factor (EGFR), EGFRvIII. The EGFRvIII mutation is reported in 25–30% of GBM and was thought to be an independent negative prognostic factor [37]. Several forms of EGFRvIII vaccines were studied in phase 1 and 2 clinical trials with promising results. This leads to the development of rindopepimut, a conjugated EGFRvIII-specific peptide (also known as CDX-110 and PEPvIII), by Celldex therapeutics. Phase 1 and 2 clinical trials demonstrated promising mOS compared to historical controls leading to an international phase 3, ACT IV, clinical trial in newly diagnosed GBM patients with EGFRvIII mutation. This randomized study of rindopepimut versus control (KLH), added to the SOC adjuvant temozolomide, showed an impressive mOS of 20.1 months in the vaccine arm. However, the control of KLH alone had 20-month mOS, far exceeding the historical controls of 15.2 months. This called into question EGFRvIII as a negative prognostic biomarker. Interestingly, loss of antigen was seen in both the treatment and control arms [38••]. A phase 2 study of rindopepimut in rGBM in combination with BEV does favor treatment with mOS of 12 months in the vaccine group compared to 8.8 months in the KLH group. This finding suggests that combination of vaccine with anti-VEGF therapy may be necessary for a single antigen vaccine to demonstrate survival benefit. At this point, the future of studies of rindopepimut is unclear; however, there are ongoing CAR-T studies targeting this tumor-specific antigen (discussed below).

A number of tumor-associated antigens are being studied in GBM, and while not specific to tumor cells, limited expression elsewhere makes these safe targets for study [39]. Another single antigen vaccine with early promise is SurVaxM, a peptide mimic of survivin conjugated to KLH. While survivin is expressed in the majority of GBM, the vaccine is HLA-restricted, limiting patient inclusion [40]. Phase 2 results of this vaccine added to the SOC adjuvant temozolomide in newly diagnosed GBM were presented at SNO 2018. While immature, mOS in this single arm phase 2 study was a promising 26 months [41]. A randomized phase 2 for new diagnosis GBM of the combination of SurVaxM with PD1 blockade is planned.

There are several other peptide vaccines targeting multiple antigens. Another recently presented vaccine, SL701, consists of short synthetic peptides targeting IL-13Ra2, ephrin A2, and survivin. This is also an HLA-A2-restricted vaccine that has been studied in the first recurrence of GBM, and was found to have a mOS of 12 months in the second phase of this study. Target-specific CD8 response seen in 8/28 patients was associated with longer survival [42].

A six synthetic peptide stimulated DC vaccine with initial promise was ICT-107. In a phase 1 trial, it was found to be safe with a suggestion of benefit to patients who were HLA-A2 positive. STING (NCT 02546102) was a phase 3 clinical trial that opened in 2016, but later suspended in 2017 due to funding. There are many other ongoing studies of vaccines targeting tumor-associated antigens in GBM using peptide, DNA with immune stimulants as well as stimulated DC cells. A review of completed vaccine trials in GBM was recently published this year by Michael Lim [39].

### Customized vaccines

For patients who have surgically accessible disease, custom vaccines are a promising area of clinical research. These vaccines require a minimum volume of resectable tumor to generate a custom vaccine, which limits the population of eligible patients.

DC-Vax-L uses whole tumor lysate to pulse patient-derived DCs. Currently over 10 years from diagnosis, some of the patients enrolled in the original phase 1 study of this vaccine are still alive [43]. The phase 3 of DC-Vax-L in newly diagnosed GBM results is still blinded; however, recent reports described a mOS of 23.1 months for all participants (90% of whom received the DC-Vax-L treatment due to crossover design). While the data remains blinded, there are concerns that this may only be interpreted as a single-arm study of 331 patients due to cross over as a result of pseudo-progression and not true progression [44•]. Again promising are reports of durable responders in the phase 3 with survival exceeding 7 years. This vaccine is also being studied in a phase 2 clinical trial in combination with the PD-1 inhibitor, nivo (NCT03014804).

Another promising custom vaccine being studied for new diagnosis GBM is HSPPC-96 (Prophage). In a single-arm phase 2 trial, mOS was 23.8 months; however, when patients were separated by PD-L1 expression on myeloid cells, mOS for those with low expression was an impressive 44.7 months [45]. HSPPC-96 is currently being studied in combination with pembro for newly diagnosed GBM. HSPPC-96 was also studied in 41 patients with rGBM with a mOS of 42.6 weeks [46]. A summary of key vaccine clinical trials in GBM is provided in Table 2.

**Table 2:** Key Vaccine Clinical Trials in GBM

Vaccine	Vaccine Category	NCT	Disease	Additional therapy	Control	Phase	n	Status	Results (treatment v. control)
Rindopepimut	Peptide	NCT01480479 (ACT IV)	new diagnosis GBM	GM-CSF + TMZ	KLH + TMZ	3	745	Terminated early (11/2016)	mOS 20.1 months v. 20.0 months
		NCT00458601 ACT III	new diagnosis GBM	GM-CSF + TMZ	none	2	82	Completed 5/2016	mOS 21.8m
		NCT01498328 (ReACT)	recurrent GBM	GM-CSF + BEV	KLH + BEV	2	127	Completed 2015	mOS 12.0m v. 8.8m
DC-Vax	DC vaccine autologus tumor pulsed	NCT00045968	new diagnosis GBM		unpulsed PBMC	3	348	Active, not recruiting	
		NCT03014804	recurrent GBM	Nivolumab	vaccine alone	2	30	planned to open 1/2019	
		NCT02146066	expanded access for patients who sceen-failed						
HSPPC-96	Custom Peptide	NCT03018288	new diagnosis GBM (MGMT -, IDH wt)	pembrolizumab + TMZ	pembrolizumab + TMZ	2	108	Ongoing	
		NCT00905060	new diagnosis GBM	TMZ	none	2	46	completed	mOS 23.8m
		NCT00293423	GTR recurrent GBM	TMZ	none	2	41	completed	mOS 10.65m
		NCT01814813	recurrent GBM	BEV	BEV	2	90	not recruiting	
		NCT02722512	new and recurrent HGG and recurrent ependymoma 3-21 years			1	20		
pp65 DC	DC - peptide pulsed CMV antigens	NCT02465268 (ATTAC-II)	new diagnosis GBM	GM-CSF + Td +TMZ	unpulsed PBMC	2	150	recruiting planned to open	
		NCT03688178 (DERIVc)	new diagnosis GBM	variliumab	vaccine alone	2	112	1/2019	
		NCT03615404	new diagnosis GBM <18 years old	GM-CSF + Td +TMZ	none	1	10	recruiting	
		NCT00639639	new diagnosis GBM	Td + TMZ	none	1	16	completed	
		NCT02529072 (AVERT)	recurrent HGG	nivolumab		1	7	not recruiting	
ICT-107	DC -peptide pulsed	NCT01280552	new diagnosis GBM	TMZ	unpulsed DC cells	2	124	completed	mOS 18.3 v. 16.7m
		NCT 02546102	new diagnosis GBM	TMZ	Control injection	3	414	suspended due to funding	
TVI-Brain-1	DC stimulated killer T cells	NCT01290692	recurrent GBM	GM-CSF		2	86	completed 2/2014	Company website notes patient 5 years survivor and mOS 50% greater than historical controls

*GTR* gross total resection, *rGBM* recurrent GBM, *TMZ* temozolomide, *BEV* bevacizumab, *nivo* nivolumab, *pembro* pembrolizumab, *mOS* median overall survival, *Td* Tetanus-Diphtheria Toxoid, *DC* dendritic cell, *KLH* Keyhole limpet hemocyanin, *HGG* high grade glioma

## Vaccine biomarkers

At this time, tumor-associated and tumor-specific antigen vaccines require confirmation that the tumor expresses the targeted antigen. Additionally, many tumor antigens are restricted to specific HLA types (class I restricted cytotoxic T cell or class II restricted helper T cell epitopes), for example HER-2, IL13Ra2, MAGE-1, and survivin. For these tumor-associated antigen vaccines, effective vaccines require tumor antigens that are presented on these restricted HLA alleles to generate an immune response [47]. This narrows the generalizability of these vaccines and clinical trials restrict enrollment to patients with the specific HLA alleles of interest, or stratify results based on HLA type.

Outside of antigen expression and HLA subtyping, there are not prospective biomarkers for response to GBM vaccines. All vaccine clinical trials are looking for correlates to predict immune response and hopefully survival response. Some of those that have been examined include IL-12 production [48], myeloid PD-L1 expression [45], T-reg CTLA-4 expression [49], circulating exomes [50, 51], and antibody production and CD8 response [52].

Additionally, as the DC-Vax-L clinical trial has demonstrated, our current imaging markers have undergone dramatic revolution in the past 10 years limiting the use of MRI for determining progression-free survival as a clinical trial outcome [53•].

## CAR-T

CAR-T cell therapy is a newer therapy in oncology, currently approved in B cell lymphoma and leukemia [54]. In brief, CAR-T cells are autologous or allogeneic T cells modified such that the extracellular domains recognizes a unique tumor-associated antigen and the intracellular domain contains a T cell activation signal [55•]. These modified T cells are then administered into the patient, where they can initiate targeted lysis of cells bearing the associated tumor antigen [56]. CAR-T cell therapy has the advantage of bypassing the need for MHC presentation of antigen and development of adaptive immune response as well as bypassing the need for co-stimulatory signals.

Given the success with B cell lymphomas and leukemias, the potential of CAR-T cell therapy for solid tumors, including GBM, has been undergoing investigation [57]. To date, GBM patients treated with CAR-T cell therapy have not had unmanageable CNS effects, a concern given the side effect of elevated intracranial pressure and associated encephalopathy seen in CAR-T cell therapy in B cell lymphoma [58]. The tumor antigens that have been most investigated for CAR targets in GBM to date are IL-13Ra2, EGFRvIII, and Her2.

Interleukin 13 receptor alpha 2 (IL-13Ra2) modulates activation along the rapamycin pathway, and is typically associated with worsened prognosis in GBM [59, 60]. A safety and efficacy trial of CAR T cell therapy in GBM with IL-13Ra2 as the tumor marker was performed on a group of three patients who had a post-resection intracranial infusion, followed by a trial of post-resection direct intratumoral infusion followed intraventricular infusion [61, 62•]. One patient had a dramatic response with clinical and radiographic response that lasted for 7.5 months; however, his disease ultimately did recur [62•]. This CAR-T target is continuing to be studied in intratumoral, intraventricular, and dual delivery systems.

EGFRvIII, as described above in the vaccine section, has also been used a CAR-T cell therapy target. A study regarding tumor infiltration of CAR-T cell performed on ten patients who were given a single peripheral CAR-T cell infusion [63]. Brain specimens in the two patients who underwent post-infusion resection showed increased intratumoral EGFRvIIICAR-T DNA compared to peripheral blood after 2 weeks, and most patients showed decreased expression of EGFRvIII in tumors resected after infusion, suggesting infiltration of the CAR-T cells into the tumor. Patients did not experience tumor regression, although it is notable that this was a particularly poor prognostic group (MGMT negative and most with multifocal disease).

Human epidermal growth factor receptor 2 (HER-2), a tyrosine kinase receptor with high expression in some forms of GBM, has also been used as a potential target [64, 65]. Peripheral infusion of virus-specific (CMV seropositive) HER-2 CAR-T cells demonstrated relative safety of this method as well as persistence of HER-2 CAR-T cells over time (measured for 1 year) [66•]. Seven of the 17 patients did have a period of 8 weeks to 29 months of stable disease and one patient had partial response [66•].

There are currently several active trials investigating the range of intracavitary, intraventricular, and intravenous modes of administration of CAR-T cells for the antigens above as well as EphA2 (NCT02575261). Current studies suggest encouraging results regarding safety and penetrance of CAR-T cells into GBM, although findings regarding effect on tumor growth and recurrence are less conclusive.

No clear biomarkers for treatment response have been established for CAR-T in GBM. In other malignancies, mechanisms of response and resistance have been studied. It is reasonable to consider that similar biomarkers will be found for CAR-T for GBM [67–69]. Given at this time, CAR-T cell therapy is targeting single antigens, antigen escape will likely limit effectiveness of CAR-T monotherapy.

## Viral therapy

Viral therapy, while initially designed as a mechanism of gene delivery to provide tumor cells with susceptibility to chemotherapy, is now recognized as a form of immunotherapy. Infection of tumor cells with virus attracts the innate immune system leading to cytokine release and tumor cell lysis. This promotes generation of an adaptive immune response to new tumor antigens and potentially development of a long-term immunotherapy effect [70]. While no proven survival benefit has been shown, the excitement about this therapy is largely driven by the population of long-term survivors which was recently reviewed [12•].

Two of these therapies have made it to phase 3 clinical trials, ASPECT and Toca5. The first, ASPECT, studied a replication defective adenovirus, sitimagene ceradenovec, in newly diagnosed GBM [71]. This clinical trial enrolled during early 2000s when the neuro-oncology community was transitioning to the current SOC of adjuvant TMZ. As a result, not all patients within the trial were treated with what is now considered SOC. While there was prolonged time to death or re-intervention in the patients treated with virus, there was no difference in mOS.

The Toca5 clinical trial is comparing Toca-511 (a non-lytic retrovirus expressing cytosine deaminase) to standard therapies for recurrent high grade gliomas. This trial recently completed enrollment; however, phase 3 data is not yet available. If the patients in the phase 1 study were narrowed to patients who would have been eligible for the phase 3, there were 5 patients out of 23 who had durable responses (defined as greater than 24 weeks) and as of August 2017, all of those patients were still alive, one over 4 years [72].

Several other viral therapies have reported GBM patients with durable responses [12•]. These include replication competent HSV1 (G207), parvovirus (ParvOryx01), adenovirus (DNX-2401), and poliovirus (PVSRIPO). Long-term survivors are also reported in another adenovirus that functions as means of gene delivery to allow local delivery of IL-12 [73].

Ongoing study of most of these viruses is now including safety for the combination of viral delivery with checkpoint inhibition.

## Conclusion

The success of immunotherapy in GBM faces several obstacles including the highly immunosuppressive nature of GBM and the limitations of the immune response in the central nervous system. Learning from phase 3 clinical trial failures, the future of immunotherapy for GBM appears most hopeful for combination therapies driven by biomarkers for appropriate patient selection. Given the extreme need for improved survival in GBM, current clinical trials are evaluating checkpoint inhibition in combination with novel therapies including vaccines, CAR-T cell therapy, and viral therapy.
